# Colorectal cancer prognosis based on dietary pattern using synthetic minority oversampling technique with K-nearest neighbors approach

**DOI:** 10.1038/s41598-024-67848-3

**Published:** 2024-07-31

**Authors:** S. Thanga Prasath, C. Navaneethan

**Affiliations:** School of Computer Science Engineering and Information Systems, Vellore Institute of Technology, Vellore, Tamil Nadu India

**Keywords:** Colorectal cancer prognosis, Dietary, Dairy products, Machine learning (ML), Synthetic minority oversampling technique-SMOTE, KNN algorithm (KNN), Health care, Risk factors

## Abstract

Generally, a person’s life span depends on their food consumption because it may cause deadly diseases like colorectal cancer (CRC). In 2020, colorectal cancer accounted for one million fatalities globally, representing 10% of all cancer casualties. 76,679 males and 78,213 females over the age of 59 from ten states in the United States participated in this analysis. During follow-up, 1378 men and 981 women were diagnosed with colon cancer. This prospective cohort study used 231 food items and their variants as input features to identify CRC patients. Before labelling any foods as colorectal cancer-causing foods, it is ethical to analyse facts like how many grams of food should be consumed daily and how many times a week. This research examines five classification algorithms on real-time datasets: K-Nearest Neighbour (KNN), Decision Tree (DT), Random Forest (RF), Logistic Regression with Classifier Chain (LRCC), and Logistic Regression with Label Powerset (LRLC). Then, the SMOTE algorithm is applied to deal with and identify imbalances in the data. Our study shows that eating more than 10 g/d of low-fat butter in bread (RR 1.99, CI 0.91–4.39) and more than twice a week (RR 1.49, CI 0.93–2.38) increases CRC risk. Concerning beef, eating in excess of 74 g of beef steak daily (RR 0.88, CI 0.50–1.55) and having it more than once a week (RR 0.88, CI 0.62–1.23) decreases the risk of CRC, respectively. While eating beef and dairy products in a daily diet should be cautious about quantity. Consuming those items in moderation on a regular basis will protect us against CRC risk. Meanwhile, a high intake of poultry (RR 0.2, CI 0.05–0.81), fish (RR 0.82, CI 0.31–2.16), and pork (RR 0.67, CI 0.17–2.65) consumption negatively correlates to CRC hazards.

## Introduction

Cancer is a well-known and significant global health issue spurred on by the body’s aberrant cell multiplication and development^1,2^. According to the World Health Organization (WHO), cancer is the second leading cause of mortality in the globe, accounting for about 19.3 million new cases and 10 million deaths in 2020^3^. The International Agency for Research on Cancer (IARC) report states that this disease affects nearly everyone on earth, which means that 1 in every 5 individuals will be infected by cancer within their lifespan. 1 in every 8 males and 1 in every 11 females die from this illness^4^. This disease is generally caused by genetics, diet, lifestyle, or the environment. Whereas living in a modern and sophisticated technologically developed society, cancer incidence and mortality rates continue to climb. Medical experts forecast that cancer incidence will increase from 19.3 to 28.4 million by 2040 worldwide^5,6^. Cancer has surpassed cardiovascular disease in many countries as the leading cause of death^7,8^. As estimated by the American Cancer Society (ACS), the number of cancer patients in the United States will increase from 1.9 million in 2022 to 1.95 million in 2023, and cancer fatalities will rise from 6,09,360 in 2022 to 6,09,820 in 2023^9–11^.

Colorectal cancer (CRC) is the most common cancer in men and women. Around 2 million cases of colon cancer were diagnosed worldwide in 2020 alone and caused 1 million deaths. Globally, CRC is the third most common cancer type and the second most common cause of cancer-related deaths^12–14^. According to the ACS estimates, 1,53,020 new cases of colon cancer will be reported in the United States in 2023, resulting in 52,550 deaths^15^. CRC affects individuals depending on their diet habits and lifestyle. Almost 50% of CRC cases would be prevented by maintaining a healthy diet and regular physical activity^16^. Excess red and processed meat consumption in our daily diet doubles the risk of developing colon cancer. Numerous studies have proved that eating 100–120 g of red meat per day increases the likelihood of CRC by up to 24%^17–21^. IARC classifies red and processed meat consumption as one of the significant risk factors (Group 1 and 2A) that cause CRC cancer^22^. Therefore, CRC could be prevented by eating a nutritious diet and staying physically active^23^.

Consuming nutritious foods can help prevent colorectal cancer. Studies demonstrate that diets heavy in vegetables, dairy products, and fibre minimize the risk of CRC; instead, excessive intake of red and processed meat enhances the risk. Considering the complexities of dietary effects, it is important to examine the combined impact of mixed foods thoroughly. The present cohort study seeks to investigate the association between daily and weekly food intake patterns and CRC risk. In particular, the investigation focuses into not just general food categories like beef, chicken, pork, and dairy products, but also possible subtypes including hamburger, lean beef, dark chicken, white chicken, and value-added dairy products. This approach allows a person to choose their meal without hesitation, consume in moderation, and live healthily.

### Machine learning

The significant prevalence of obesity in today’s modern culture has made hospitals a frequent destination for most people. Cancer is a chronic disease that may impact anyone at any moment. Diagnosing deadly diseases like cancer is a very challenging and time-consuming task for physicians working in diagnostics. Early detection and treatment increase the chance of a cure. Hence, now is the time to make some slight changes to traditional medical procedures in order to make them more appropriate for the modern world to predict cancer as early as possible. Developing an effective prediction model with fast-emerging AI technologies like ML and DL can help prompt cancer detection and considerably reduce cancer mortality^24,25^.

#### Classification

To diagnose cancer patients, we designed a multi-label classification model. Identifying ML models was thus a foremost challenge. This research analyses five classification algorithms to discover which classification model outperforms the others on a real-time dataset.

##### KNN

Classification is the process of predicting a class object for which the class label is unknown, and it also distinguishes the independent and dependent features across the dataset. K-nearest neighbors, also known as KNN, is a classifier algorithm, a supervised non-parametric learning classifier process that identifies new data based on learning data (training data), and ‘k’ refers to the closest neighbors^26–30^.

##### Decision tree

A Decision Tree (DT) algorithm is a hierarchical tree-structured non-parametric supervised method of learning. For common machine-learning issues, the DT can do classification and regression^31,32^. Generally, DT has two major points one is the decision node, and the other is the leaf node^33,34^. The decision node only makes decisions based on the cluster of branches, while the leaf node is the decision’s actual output^35,36^. Merely a few algorithms can handle large datasets and flexibly fit all features. DT is one of them. For our use cases, DT is one of the best fits.

##### Random forest (RF)

Random Forest combines various decision tree outputs and based on the dense forest, it had a high possibility of improving accuracy^37–39^. RF has the technology of ensemble learning to handle multiple outputs and improve performance by solving complex problems. Surprisingly RF only takes less time to train models. One key feature of RF is that it can prevent model overfitting^40^, which is the major issue in our healthcare dataset^41^. RF performs multilabel classification.

##### Logistic regression

Logistic regression could make better results on classification and regression problems^42–44^. Here we didn’t use raw logistic regression. We took the method of this algorithm and customized it with our use case based on the runtime and used two different methods using logistic regression. The first is Logistic Regression with Classifier Chain (LRCC), and the second is Logistic Regression with Label Powerset (LRLC). The Classifier Chain models could arrange every chain randomly^45–47^. This chain will be an optimal ordering of the other classes, making the best performance while training the model. The core logic of Label Powerset is to transform all the labels into one unique label with the combinations found on the data. The model showed high computation complexity even for the worst case of (2^|C|). Both methods showed enhanced and optimized results than the raw logistic regression.

## Literature study

Colorectal cancer (CRC) remains a significant health concern even in today’s technologically advanced world. Several epidemiological studies have outlined the role of dietary factors in colorectal cancer incidence. Numerous studies on daily dietary patterns reveal that red and processed meat consumption is strongly connected to CRC risk. In 2020, Bradbury et al.^48^ investigated the correlation between red and processed meat consumption and CRC risk using the UK Biobank dataset, confirming that eating red and processed meat increases CRC risk. Notably, ingesting more than 54 g of red meat and 25 g of processed meat doubled the risk of CRC. Likewise, a study conducted by Feng et al.^49^ in 2021, using the UK Biobank dataset and Mehta et al.^50^ in the same year, using data from the USA and Puerto Rico, as well as Bernstein et al.^51^ in 2015, based on USA dataset, furtherly strengthened these results. All these studies collectively underscore the heightened risk of CRC associated with increased red and processed meat consumption.

Similarly, numerous studies have examined the impacts of other forms of meat, such as fish and poultry, on CRC risk. Bradbury et al.^48^, investigated poultry intake and found that eating more chicken could decrease the chance of CRC. Likewise, Wang et al.^52^ and Aglago et al.^53^ confirms that excessive eating of fish on a daily basis would reduce the CRC risk. Conversely, a study by Mejborn et al.^54^ did not support the notion that consuming poultry prevents CRC risk.

Likewise, Deschasaux-Tanguy et al.^55^, French data, and Collatuzzo et al.^56^ in the same year, using Iranian data, examined the effects of dietary intakes like dairy products. Both research found that milk, yogurt, cheese, and ice cream all negatively correlated with CRC risk. Additionally, confirmed that overall dairy product consumption had no significant connection with CRC risk. But, Collatuzzo et al.^56^ observed that excessive cream intake was associated with an increased risk of CRC. In contrast, research conducted in the same year by Alegria-Lertxundi et al.^57^ using Spanish data exhibited a positive connection between milk and milk product intake, particularly high-fat cheeses, and CRC risk.

Overall, these reliable results emphasize the importance of dietary choices in determining CRC risk. However, the disparities in findings among studies underscore the need for more research better to understand the complicated link between food and CRC.

## Methods and materials

### Study population identification and recruitment

The National Cancer Institute (NCI) and Cancer Data Access System (CDAS), which the US government controls collected the Prostate, Lung, Colorectal, and Ovarian (PLCO) dataset, which greatly aided this research finding. The PLCO dataset was collected as part of a cancer screening study, a kind of controlled and randomized trial. This trial’s goal is to ascertain whether or not the screening methods significantly reduce PLCO cancer mortality. This PLCO trial was conducted from 1993 to 2001. Nearly 1,55,000 males and females between 55 and 74 years of age were identified, separated into either Control or Intervention arms, and researched at ten hospitals. Participants’ cancer diagnosis and mortality details were modified in 2009 (11.3 years of follow-up) and 2018 (9.2 years of follow-up), respectively. If the participant was under 60 age or over 74 age, had a history of PLCO cancer, had both ovaries removed, and were participating in another cancer trial, they were exempted from this trial. The human subjects review boards of the National Cancer Institute (NCI) and each screening center approved this PLCO Screening Trial, and all methods were performed according to the appropriate regulations and guidelines. Furthermore, all individuals provided written informed consent.

### Questionnaire

All participants were asked three types of questions, and data were collected. Baseline Questionnaire (BQ) asked all participants for basic information such as age, occupation, smoking, drinking, and medical history, and 96.8% of participants completed it. All participants were encouraged to complete the Diet History Questionnaire (DHQ) to collect information on their daily food intake. These DHQs provide information about the daily grams and daily frequencies of various foods and beverages consumed by participants on a daily basis, and 77% of participants completed the DHQ. Only intervention arm participants were provided with the Dietary Questionnaire (DQX). And, like the DHQ, it will also contain basic details of the questionnaire, like daily foods and frequency of food consumption. These raw data are then systematically processed and converted into variables suitable for analysis, such as gram intake and daily food frequency.

### Dietary exposure assessment

All participants were questioned about alcohol servings and serving sizes, and obtained values were converted into grams using DietCalc software. Regarding food values, DietCalc was used to compute DHQ nutritional values based on food frequency, serving size, and other questionnaire data. Whereas food frequency values reflect how frequently a person consumes the food without concern for the portion size of that particular food. Sometimes, when calculating gram amounts of certain foods and food frequencies, more than one food response contributed. In such cases, a specific food’s gram or frequency values are calculated by adding the grams or frequency of all the food items together.

### Cancer ascertainment

During the PLCO trial, participants’ colorectal cancer was confirmed through Medical Record Abstraction (MRA), self-reports, family reports, and death certificates. If any confirmed invasive tumors, in situ cancers, or borderline malignancies have been identified during an annual cancer screening, they are considered the cancer endpoint. In certain situations, when clear MRA records were not accessible, existing and/or available medical records were analyzed and summarized. Also, follow-up activity continued when documentary records were not public. Most importantly, if the MRA approach finds no confirmation of colorectal cancer diagnosis, even if records confirming colorectal cancer in self-reports, family reports, and death certificates exist, they are not considered or documented as confirmed colorectal cancer evidence.

### Statistical analysis

The proposed prospective cohort study’s primary objective is to develop a precise ML model to determine the association between colorectal cancer risk and participants’ daily dietary habits. It also investigates how daily consumed food accelerates colorectal cancer. The colorectal cancer prediction model was designed using SMOTE technique and the KNN algorithm. Furthermore, risk ratio (RR) and 95% confidence interval (CI) values were used to determine cancer hazards.

The food consumption parameters of the participants are ranked separately into alcohol, beef, butter, cheese, milk, yoghurt, chicken, fish, and pork consumption values as per the research needs. For research objectives, the aforementioned participants’ food consumption values were converted into grams and utilized. Following that, study compared participants’ daily food consumption value (g) and frequency of intake of a specific food per week with CRC cancer incidence and classified the findings by food category.

The research identifies which types of food intake increase colorectal cancer threats by analyzing statistical data on daily food consumption and frequency values with research outcomes. The research outcomes are grouped into two different food types: vegetarian and non-vegetarian. The findings classify potential causes of cancer into five groups:

- *Positive relations* These foods exhibit a significant positive association with CRC risk. Output values of daily intake of specific food had a positive relationship, whereas the output values of food frequency also had a positive relationship. Such foods have been discussed in this section.
- *Negative relations* These foods are negatively related to CRC. Output values of daily intake of specific food had a negative link, as well as the output values of food frequency also had a negative connection. Such foods have been discussed in this section.
- *No positive relation* These foods have not been positively linked to CRC hazards. Let us consider that the participants consume a particular food. The output values of the participant’s daily food intake have a positive relationship, whereas the output values of the participants’ frequent food intake have a negative relationship. Similarly, the output values of the participants’ daily food intake showed a negative correlation, but the output values of the participants’ frequent food intake had a positive association. In such an uncommon circumstance, our research considered to be that these dietary values had no positive relation with CRC cancer.
- *No negative relation* These foods have also not been positively linked to CRC threat. The output values of the participant’s daily food intake have a negative relationship, and the frequency of food intake has insufficient values. In such a situation, our research considered these food items do not cause CRC threats and considered them as having no negative relation.
- *Moderate relation* Some foods are neither healthy nor hazardous for our health. That is, there is no substantial positive or negative link between that specific food and cancer. This section provides information about such foods.

#### Multilabel accuracy prediction

Calculating the accuracy of multilabel classification is a bit challenging and tricky. Determining the appropriate level of accuracy for each of these models adds complexity to the analysis. A generic function is essential to provide accuracy for all methods. The set of predicted labels in y_pred must match the corresponding set in y_true. Finally got the mean value of the y_pred and y_true arrays and returned it. This process would be handled in multiple phases, repeated according to the label size.

Adopting a healthy diet and lifestyle may reduce the chance of developing colorectal cancer. A detailed analysis of food, its quantity and frequency of intake is essential to lessen the CRC hazards. This research applies the SMOTE approach to all ML models to eliminate the data imbalances in the PLCO dataset and improve the model’s prediction. From the experimental results, it was proved that KNN-SMOTE showed enhanced accuracy than the rest of the ML models. The primary aim of the study subsequent to the implementation of KNN-SMOTE on the dataset is as follows:

- To investigate the correlations between red/white meat consumption and CRC incidence.
- To scrutinize how a certain food in a specific amount and certain frequency affects CRC likelihood.
- To research how a particular food combination would increase or decrease the CRC risk.

#### Risk ratio (RR) and confidence interval (CI)

The risk associated with the particular food intake in specific quantity (grams) and particular frequency is evaluated using Risk Ratio (RR) and 95% Confidence Interval (CI).

1 $$ {\text{Risk}}\;{\text{ratio}} = \frac{Probabability\;of\;outcome\;in\;exposed\;group}{{Probablity\;of\;outcome\;in\;unexposed\;group}} $$

The Confidence Interval (CI) measures the level of certainty or uncertainty around the relationship between CRC and food intake (g). A 95% CI is a statistical metric that provides a level of confidence of 95% about the estimated range of certainty and uncertainty.

The 95% CI is calculated as:

2 $$ {\text{CI}} = {\text{sample}}\;{\text{mean}}\; \pm \;{\text{margin}}\;{\text{error}}{.} $$

The CI is completely dependent on RR. If RR is < 1, then there is no risk of CRC. If RR = 1, then there may or may not be a risk associated with CRC in consuming the food items. If RR > 1, then there is a high-risk factor associated with CRC.

#### Dataset

The NCI and CDAS provide us with real-time datasets. To the best of our knowledge, the study further affirms that the utilized NCI dataset is the most up-to-date version available. The dataset has 231 input characteristics, two output features, and about 1,55,000 records of the participants. It has 2359 cancer records. Cancer patients constitute only 2% of the whole dataset.

#### Preprocessing

There was a lot of background noise in the dataset. Some features were of no use for training in ML models. Therefore, such columns were removed to decrease the model training time and increase the effectiveness. Since we already had an “age level” column, we decided to remove the “age” column. PLCO_id is only a patient’s unique identifier and can’t be used in model training in any way. Subsequently, duplicates and erroneous values in dietary information were meticulously identified and eliminated, along with columns having blank, null, or negative entries. A total of 27 fields were removed from the original dataset, making 204 features available for further processing. ML models cannot handle multiple data types within a single column. All rows with data of different types in the same column were evaluated carefully, and identical rows were created where necessary.

#### Usage of synthetic minority oversampling technique (SMOTE)

The real-time datasets help to quickly train our model with different ML classifiers. The model consistently achieves a validation accuracy of 95%. The reason behind this magical accuracy was that there would be limited negative or positive data. For instance, the corona fever death dataset. Corona deaths were only 4%–5%. So, while feeding this data into the ML model, it’ll learn 95% false and 5% true information. Once the fitting mechanism has completed its execution, it becomes necessary to compute the model’s accuracy. It is important to note that the model is only based on 5% of the true data. The model consistently provides erroneous accuracy rates. Those issues were called “overfitting” with a real-time dataset. This dataset has an uneven distribution of values. Therefore, we need to ensure that the dataset is well-balanced.

The SMOTE method is used to solve this problem. The primary goal of this SMOTE method was to use various KNN methods to rebalance the original dataset. The process of transforming the imbalanced dataset into a balanced dataset was accomplished using two techniques. Those were oversampling and undersampling. Undersampling simply reduces a lot of valuable data from the dataset. This could eliminate most of the valuable data without any measurement. So, this method will not be valid for our use case. The Oversampling approach is another option for SMOTE. This would create a synthetic dataset that matches our original dataset to achieve balance.

## Result and discussion

The primary goal of the research is to compare which foods are known to cause CRC cancer and which food consumption raises CRC risks. Finally, the proposed model compares the values of various food types intake and CRC cancer incidence. This investigation recommends people who read the research findings be cautious when choosing foods. And the study’s findings are not intended to frighten people.

### Performance appraisal

The proposed model is trained using five ML algorithms namely KNN, RF, DT, LRCC and LRLP. The performance of the classifiers is evaluated based on the accuracy of the employed ML models before and after applying SMOTE.

#### Accuracy comparison

Table 1 shows the accuracy comparison for all the methods before and after applying the SMOTE. From Table 1, it would be obvious that KNN performed better in classification accuracy after applying SMOTE. Thus, the study used KNN as the ML model for training the dataset to identify CRC risk factors.

**Table 1:** Accuracy comparison.

Classifiers	Before applying SMOTE	After applying SMOTE
KNN	98.43	85.18
Decision tree	96.96	84.19
Random forest	98.49	90.66
LR with classifier chain	98.47	59.78
LR with label powerset	98.48	61.13

#### Performance metrics evaluation for KNN

**Figure 1:**
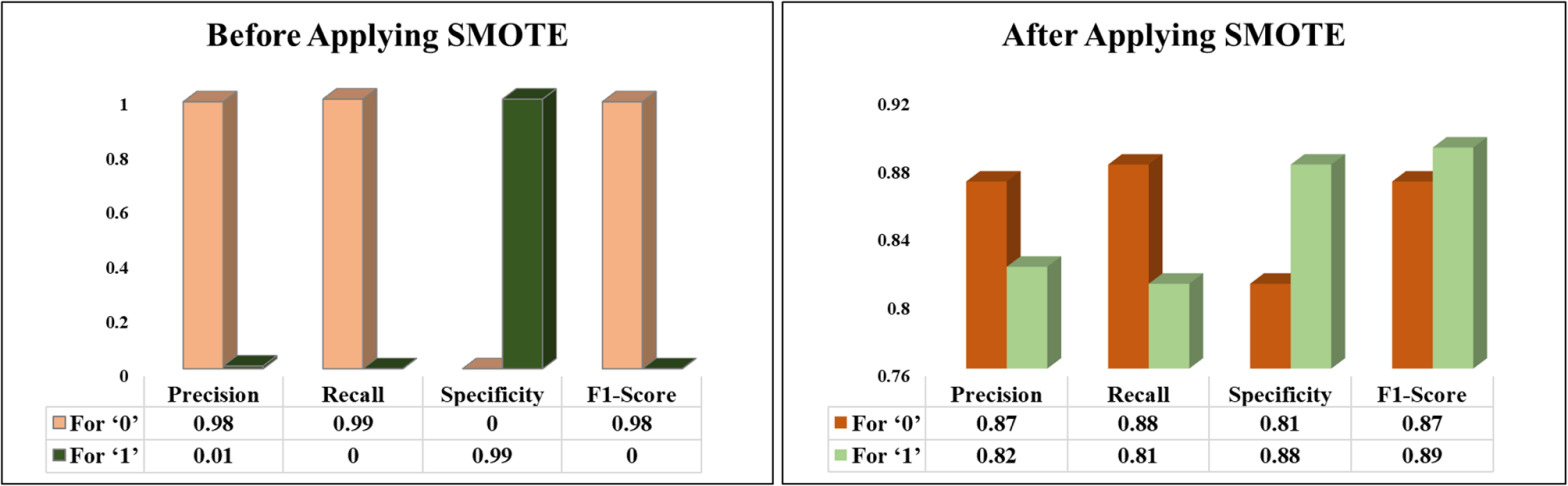
Performance comparison of KNN algorithm.

The proposed KNN algorithm’s effectiveness is measured using precision, recall, specificity, and F1-score. Table 1 shows that our ML model achieved 98.43% accuracy before employing SMOTE. Fewer data points seemed to have the value ‘1’. Hence, the proposed model is erroneously assumed and trained. During the accuracy calculation, the classifier misdiagnosed each parameter as a ‘0’ value. This is the reason for the extremely high accuracy before using SMOTE. After applying SMOTE, the study obtained an 85% success rate. At the same time, the model performed even better when identifying cancer patients from healthy participants. Consequently, the proposed SMOTE-KNN algorithm would correctly predict cancerous and non-cancerous individuals concerning food quantity and frequency. A visual representation of the efficiency metrics used to evaluate the proposed model is shown in Fig. 1.

### Result analysis

The primary goal of the research is to compare which foods are known to cause CRC cancer and which food consumption raises CRC risks. Additionally, the study compares intake levels of various food types with CRC cancer incidence. This investigation recommends people who read the research findings be cautious when choosing foods. And the study’s findings are not intended to frighten people.

Research outcomes have been described in two different sections.

*Section 1* Based on the relationship between food consumption value, frequency, and CRC cancer incidence, the type of food that stimulates CRC risk will be identified.

*Section 2* The study addressed which kind of food combinations (mixed foods) increase the CRC risk.

Therefore, we estimated the risk ratio and 95% confidence interval in all sections to classify the risk factors.

#### Daily food consumption and food frequency responses

This section compares the value of the participants’ daily food consumption with the values of how frequently they consume those foods each week and discusses the risk values of the specific foods. Serving size is used to represent the food intake values of participants. Based on total food consumption, the serving size values for each food fluctuate. Table 2 uses the following values for serving size: One serving size of liquor was 100 g. One serving size of beef, fish, pork, and poultry was 74 g. One serving size of butter, cheese, and ice cream was 10 g. One serving size of milk and yogurt was 125 g. Furthermore, Table 2 shows the risk ratio based on daily food consumption, whereas Table 3 displays the risk ratio for weekly food consumption.

**Table 2:** CRC risk based on daily food intake and serving size.

Positive relation										
Food items servings per day	RR	CI	RR	CI	RR	CI	RR	CI	RR	CI
Alcohol^a^	1		2		3		4		5	
Beer	0.91	0.81–1.02	0.96	0.80–1.16	1.18	0.90–1.56	1.18	1.00–1.40	–	–
Liquor	0.73	0.55–0.97	1.17	0.77–1.78	1.61	1.08–2.39	–	–	–	–
Beef^b^	1		2		3		4		5	
Stew	0.92	0.69–1.23	1.02	0.72–1.45	1.24	0.69–2.23	1.32	0.43–4.06	–	–
Beef roast	0.82	0.46–1.48	1.22	0.68–2.19	–	–	–	–	–	–
Roasted beef with sandwich	0.96	0.31–2.97	1.04	0.34–3.19	–	–	–	–	–	–
Butter^c^	1		2		3		4		5	
Reduced fat butter with bread	0.5	0.23–1.10	1.99	0.91–4.39	–	–	–	–	–	–
Reduced fat butter with potatoes	0.37	0.10–1.45	2.68	0.69–10.42	–	–	–	–	–	–
Reduced fat butter with vegetables	0.56	0.08–3.90	1.77	0.26–12.27	–	–	–	–	–	–
Cheese^c^	1		2		3		4		5	
Regular cream cheese	0.9	0.55–1.47	1.11	0.68–1.81	–	–	–	–	–	–
Milk^d^	1		2		3		4		5	
1% Milk with cereal	0.98	0.71–1.34	0.99	0.71–1.38	1.48	0.56–3.91	–	–	–	–
Milk rice with cereal	0.41	0.06–2.81	2.43	0.36–16.65	–	–	–	–	–	–
Soy milk with cereal	0.98	0.32–3.01	1.02	0.33–3.14	–	–	–	–	–	–
Whole milk with cereal	0.81	0.53–1.24	1.24	0.81–1.89	–	–	–	–	–	–
Mixed foods^b^	1		2		3		4		5	
Chili	0.75	0.50–1.11	1.39	0.88–2.20	1.25	0.47–3.29	1.11	0.28–4.41	–	–
Meat component (stews/pot pies/mixtures)	0.42	0.14–1.28	2.37	0.78–7.22	–	–	–	–	–	–
Meat component (chili)	0.61	0.09–4.22	1.64	0.24–11.38	–	–	–	–	–	–
Meat component (ground beef meatballs/loaves/mixtures)	0.95	0.60–1.50	1.06	0.67–1.68	–	–	–	–	–	–
Negative relation										
Beef^b^	1		2		3		4		5	
Beef steak	1.13	0.64–1.99	0.88	0.50–1.55	–	–	–	–	–	–
Butter^c^	1	2	3	4	5					
Regular butter with bread	1.17	0.80–1.72	0.86	0.58–1.26	–	–	–	–	–	–
Regular butter with vegetables	1.9	0.48–7.57	0.53	0.13–2.09	–	–	–	–	–	–
Cheese^c^	1		2		3		4		5	
Low fat cheese	1.12	0.86–1.46	0.9	0.67–1.22	0.98	0.49–1.96	0.8	0.26–2.48	0.68	0.22–2.09
Nonfat cheese	1.37	0.82–2.26	0.88	0.51–1.52	0.35	0.09–1.39	–	–	–	–
Cheese sauce	1.3	0.78–2.15	0.84	0.48–1.47	0.59	0.19–1.82	–	–	–	–
Milk^d^	1		2		3		4		5	
2% Milk not with coffee, tea, or cereal	1.04	0.88–1.22	0.98	0.76–1.26	0.99	0.75–1.30	0.92	0.68–1.24	–	–
Nonfat/skim milk with cereal	1.18	0.92–1.51	0.85	0.66–1.09	–	–	–	–	–	–
Nonfat/skim milk with coffee and tea	3.52	0.88–14.0	0.28	0.07–1.13	–	–	–	–	–	–
Nonfat/skim milk not with coffee, tea, or cereal	1.18	1.04–1.34	0.9	0.71–1.14	0.81	0.66–1.00	0.88	0.73–1.07	–	–
Soy milk not with coffee, tea, or cereal	3.71	0.52–26.21	0.27	0.04–1.91	–	–	–	–	–	–
Yogurt^d^	1		2		3		4		5	
All yogurt	1.33	1.09–1.62	0.82	0.66–1.01	0.58	0.36–0.93	0.36	0.05–2.57	–	–
Frozen yogurt, ices, sorbet	1.8	0.94–3.46	0.51	0.24–1.07	0.8	0.20–3.18	–	–	–	–
Chicken^b^	1		2		3		4		5	
Chicken in mixtures	1.23	0.96–1.58	0.86	0.67–1.11	0.44	0.17–1.17	–	–	–	–
Chicken dark meat	1.51	0.21–10.61	0.66	0.09–4.66	–	–	–	–	–	–
Chicken not in mixtures	4.94	1.24–19.74	0.2	0.05–0.81	–	–	–	–	–	–
Chicken white meat	4.21	0.59–29.79	0.24	0.03–1.68	–	–	–	–	–	–
Ground chicken/turkey	1.14	0.16–8.01	0.88	0.12–6.14	–	–	–	–	–	–
Turkey	2.3	0.33–16.24	0.43	0.06–3.07	–	–	–	–	–	–
Fish^b^										
Fish not fried/no fat added	1.23	0.46–3.25	0.82	0.31–2.16	–	–	–	–	–	–
Canned or water packed tuna	1.1	0.28–4.35	0.91	0.23–3.61	–	–	–	–	–	–
Tuna	1.37	0.35–5.44	0.73	0.18–2.90	–	–	–	–	–	–
Mixed foods^b^										
Hot dogs	1.46	0.21–10.28	0.68	0.10–4.81	–	–	–	–	–	–
Lasagna, ravioli, shells	1.48	0.82–2.67	0.68	0.36–1.31	0.64	0.16–2.53	–	–	–	–
No positive relation										
Beef^b^	1		2		3		4		5	
Roasted beef not with sandwich	1.01	0.42–2.42	0.99	0.41–2.36	–	–	–	–	–	–
Beef burgers	0.94	0.52–1.69	1.07	0.59–1.92	–	–	–	–	–	–
Lean beef burgers	0.21	0.05–0.78	0.58	0.15–2.30	–	–	–	–	–	–
Regular beef burgers	0.81	0.26–2.49	1.23	0.40–3.78	–	–	–	–	–	–
Ground beef meatballs	0.95	0.60–1.50	1.06	0.67–1.67	–	–	–	–	–	–
Lean beef steak	0.75	0.36–1.56	1.33	0.64–2.78	–	–	–	–	–	–
Butter^c^	1		2		3		4		5	
Regular butter with bread	0.77	0.41–1.42	1.31	0.71–2.41	–	–	–	–	–	–
Cheese^c^	1		2		3		4		5	
Cheesecake	0.98	0.80–1.20	1.03	0.84–1.26	–	–	–	–	–	–
Low fat cheese cream	0.54	0.27–1.07	1.85	0.93–3.67	–	–	–	–	–	–
Milk^d^	1		2		3		4		5	
1% Milk with coffee and tea	0.42	0.20–0.86	0.98	0.14–6.86	2.74	1.05–7.16	4.53	1.19–17.26	–	–
1% Milk not with coffee, tea, or cereal	0.99	0.83–1.19	0.94	0.68–1.30	1.02	0.74–1.40	1.06	0.78–1.43	–	–
2% Milk with cereal	1	0.79–1.26	1	0.80–1.26	–	–	–	–	–	–
2% Milk with coffee and tea	2.16	0.70–6.68	0.46	0.15–1.43	–	–	–	–	–	–
Condensed milk with coffee and tea	0.24	0.04–1.63	4.1	0.62–27.40	–	–	–	–	–	–
Whole milk with coffee and tea	1.76	0.44–7.00	0.57	0.14–2.26	–	–	–	–	–	–
Chicken^b^	1		2		3		4		5	
Non fried skinless chicken dark meat	0.4	0.06–2.71	2.53	0.37–17.27	–	–	–	–	–	–
Fried chicken white meat with skin	0.44	0.06–3.03	2.86	0.42–19.42	–	–	–	–	–	–
Fish^b^	1	2	3	4	5					
Fat added fried fish	1.29	0.54–3.08	0.78	0.32–1.86	–	–	–	–	–	–
Pork^b^	1		2		3		4		5	
Pork	1.5	0.38–5.96	0.67	0.17–2.65	–	–	–	–	–	–
No negative relation										
Butter^c^	1		2		3		4		5	
Regular butter with potatoes	1.1	0.57–2.10	0.91	0.48–1.75	–	–	–	–	–	–
Cheese^c^	1		2		3		4		5	
Regular cheese	1.07	0.94–1.23	0.93	0.78–1.10	0.74	0.49–1.14	0.96	0.69–1.35	–	–
Moderate relation										
Low fat ice cream^c^	0.95	0.82–1.10	1.08	0.88–1.31	1.09	0.83–1.45	0.58	0.30–1.10	1.13	0.83–1.54
Regular ice cream^c^	0.92	0.83–1.02	1.04	0.90–1.21	0.98	0.81–1.19	1.31	1.03–1.65	1.12	0.91–1.39

‘–’, Not applicable (no data/studies available to calculate RR, CI).

^a^This value refers to a serving (100 g).

^b^This value refers to a serving (74 g).

^c^This value refers to a serving (10 g).

^d^This value refers to a serving (125 g).

**Table 3:** Association between food consumption frequency and CRC risk.

Positive relation														
Food items servings per day	RR	CI	RR	CI	RR	CI	RR	CI	RR	CI	RR	CI	RR	CI
Alcohol	1 Time		2 Times		3 Times		4 Times		5 Times		6 Times		More than 6 Times	
Beer	0.91	0.81–1.02	1.04	0.88–1.22	0.89	0.48–1.66	1.13	0.87–1.47	1.05	0.70–1.58	1.82	0.88–3.78	1.17	0.94–1.45
Liquor	1.02	0.90–1.15	0.8	0.65–1.00	–	–	1.05	0.82–1.34	1	0.71–1.41	–	–	1.17	0.95–1.44
Beef	1 Time		2 Times		3 Times		4 Times		5 Times		6 Times		More than 6 Times	
Stew	0.98	0.72–1.34	0.94	0.65–1.36	–	–	1.31	0.71–2.43	–	–	–	–	3.37	0.87–13.00
Beef roast	0.89	0.76–1.04	1.11	0.95–1.30	–	–	–	–	–	–	–	–	–	–
Roasted beef with sandwich	0.95	0.70–1.30	1.05	0.77–1.43	–	–	–	–	–	–	–	–	–	–
Butter	1 Time		2 Times		3 Times		4 Times		5 Times		6 Times		More than 6 Times	
Reduced fat butter with bread	0.93	0.74–1.17	0.92	0.64–1.32	1.49	0.93–2.38	1.03	0.56–1.92	1.23	0.52–2.93	1.17	0.44–3.09	1.01	0.55–1.88
Reduced fat butter with potatoes	0.86	0.61–1.21	1.11	0.72–1.71	1.18	0.59–2.35	–	–	–	–	–	–	–	–
Reduced fat butter with vegetables	0.56	0.08–3.90	1.77	0.26–12.27	–	–	–	–	–	–	–	–	–	–
Cheese	1 Time		2 Times		3 Times		4 Times		5 Times		6 Times		More than 6 Times	
Regular cream cheese	0.85	0.66–1.08	1.13	0.82–1.55	1.08	0.54–2.16	1.25	0.70–2.25	1.48	0.74–2.93	–	–	–	–
Milk	1 Time		2 Times		3 Times		4 Times		5 Times		6 Times		More than 6 Times	
1% Milk with cereal	0.96	0.82–1.12	1.11	0.83–1.48	–	–	1.17	0.92–1.47	1.02	0.85–1.21	–	–	–	–
Milk rice with cereal	0.56	0.21–1.47	1.79	0.68–4.70	–	–	–	–	–	–	–	–	–	–
Soy milk with cereal	0.97	0.60–1.58	0.91	0.34–2.41	–	–	1.08	0.61–1.89	–	–	–	–	–	–
Whole milk with cereal	0.81	0.65–1.01	1.42	1.00–2.02	–	–	1.13	0.86–1.48	–	–	–	–	–	–
Negative relation														
Food items servings per day	1 Time		2 Times		3 Times		4 Times		5 Times		6 Times		More than 6 Times	
Beef	RR	CI	RR	CI	RR	CI	RR	CI	RR	CI	RR	CI	RR	CI
Beef steak	1.14	0.81–1.61	0.88	0.62–1.23	–	–	–	–	–	–	–	–	–	–
Butter	1 Time		2 Times		3 Times		4 Times		5 Times		6 Times		More than 6 Times	
Regular butter with bread	1.11	1.00–1.24	0.91	0.76–1.08	0.8	0.62–1.04	0.83	0.64–1.08	0.95	0.67–1.36	0.81	0.54–1.22	1.09	0.89–1.34
Regular butter with vegetables	1.26	1.05–1.51	0.74	0.57–0.96	0.86	0.51–1.45	0.78	0.53–1.14	0.97	0.66–1.43	–	–	–	–
Cheese	1 Time		2 Times		3 Times		4 Times		5 Times		6 Times		More than 6 Times	
Low fat cheese	1.09	0.92–1.28	0.96	0.79–1.18	0.87	0.59–1.27	0.86	0.55–1.34	0.84	0.42–1.68	–	–	–	–
Nonfat cheese	1.1	0.85–1.42	1.04	0.77–1.40	0.57	0.26–1.28	0.75	0.38–1.49	–	–	–	–	–	–
Cheese sauce	1.15	0.92–1.43	0.86	0.65–1.12	0.96	0.60–1.55	0.84	0.45–1.55	–	–	–	–	–	–
Milk	1 Time		2 Times		3 Times		4 Times		5 Times		6 Times		More than 6 Times	
2% Milk not with coffee, tea or cereal	0.99	0.86–1.13	1.23	0.97–1.54	–	–	0.91	0.67–1.23	0.93	0.63–1.38	–	–	0.96	0.79–1.17
Nonfat/skim milk with cereal	1.2	1.07–1.33	0.89	0.71–1.10	–	–	0.76	0.63–0.92	0.9	0.73–1.10	–	–	0.93	0.75–1.17
Nonfat/skim milk with coffee and tea	1.08	0.88–1.33	–	–	0.76	0.43–1.34	–	–	–	–	0.96	0.77–1.20	–	–
Nonfat/skim milk not with coffee, tea or cereal	1.2	1.07–1.34	0.83	0.64–1.07	–	–	1.04	0.81–1.33	0.8	0.57–1.13	–	–	0.82	0.71–0.94
Soy milk not with coffee, tea or cereal	3.46	0.87–13.82	0.29	0.07–1.15	–	–	–	–	–	–	–	–	–	–
Yogurt	1 Time		2 Times		3 Times		4 Times		5 Times		6 Times		More than 6 Times	
All yogurt	1.38	1.19–1.61	0.7	0.55–0.89	–	–	0.82	0.64–1.05	0.93	0.65–1.32	–	–	0.5	0.32–0.78
Frozen yogurt, ices, sorbet	1.35	1.01–1.80	0.71	0.47–1.07	–	–	0.85	0.53–1.36	0.66	0.33–1.32	–	–	–	–
Chicken	1 Time		2 Times		3 Times		4 Times		5 Times		6 Times		More than 6 Times	
Chicken mixtures	1.14	0.99–1.32	0.93	0.78–1.11	–	–	0.82	0.63–1.07	0.67	0.32–1.41	–	–	0.47	0.07–3.28
Chicken not in mixtures	1.15	1.02–1.31	0.91	0.79–1.05	0.6	0.19–1.85	0.77	0.58–1.01	0.92	0.49–1.70	–	–	–	–
Ground chicken/turkey	1.11	0.70–1.77	1.1	0.65–1.86	–	–	0.56	0.18–1.72	0.51	0.07–3.61	–	–	–	–
Turkey	1.27	0.91–1.77	0.88	0.59–1.32	–	–	0.69	0.36–1.32	0.36	0.05–2.55	–	–	0.75	0.11–5.30
Fish	1 Time		2 Times		3 Times		4 Times		5 Times		6 Times		More than 6 Times	
Not fried fish and No fat added	1.51	1.15–1.97	0.63	0.46–0.86	0.77	0.47–1.26	–	–	–	–	–	–	–	–
Water packed canned tuna	1.44	1.10–1.88	0.68	0.49–0.94	–	–	0.78	0.49–1.26	–	–	–	–	–	–
No positive relation														
Food items servings per day	1 Time		2 Times		3 Times		4 Times		5 Times		6 Times		More than 6 Times	
Beef	RR	CI	RR	CI	RR	CI	RR	CI	RR	CI	RR	CI	RR	CI
Beef steak	0.91	0.62–1.34	1.13	0.74–1.71	–	–	1.21	0.46–3.20	–	–	–	–	–	–
Beef burgers	0.94	0.80–1.11	1.12	0.94–1.35	0.88	0.61–1.26	–	–	–	–	–	–	–	–
Ground beef meatballs	0.99	0.82–1.19	1.04	0.85–1.28	–	–	0.93	0.60–1.44	0.67	0.17–2.65	–	–	–	–
Lean beef steak	1.03	0.69–1.55	0.97	0.64–1.46	–	–	–	–	–	–	–	–	–	–
Butter	1 Time		2 Times		3 Times		4 Times		5 Times		6 Times		More than 6 Times	
Regular butter	1.05	0.87–1.27	1.07	0.84–1.36	0.83	0.52–1.33	0.8	0.54–1.19	–	–	–	–	–	–
Reduced fat butter with pancakes	0.49	0.21–1.17	2.03	0.86–4.81	–	–	–	–	–	–	–	–	–	–
Cheese	1 Time		2 Times		3 Times		4 Times		5 Times		6 Times		More than 6 Times	
Low fat cream cheese	0.88	0.68–1.16	1.27	0.92–1.76	0.89	0.55–1.46	–	–	–	–	–	–	–	–
Milk	1 Time		2 Times		3 Times		4 Times		5 Times		6 Times		More than 6 Times	
1% Milk with coffee and tea	1.02	0.75–1.39	–	–	1.13	0.54–2.35	–	–	–	–	1.19	0.50–2.83	0.92	0.64–1.33
1% Milk not with coffee, tea or cereal	1.04	0.89–1.23	0.97	0.68–1.39	–	–	0.73	0.47–1.14	1.02	0.83–1.24	–	–	–	–
2% Milk with cereal	0.91	0.81–1.03	1.06	0.85–1.32	1.12	0.92–1.35	1.08	0.89–1.32	–	–	–	–	–	–
2% Milk with coffee and tea	0.94	0.78–1.14	–	–	1.1	0.67–1.79	–	–	–	–	1.05	0.85–1.29	–	–
Condensed milk with coffee and tea	1.42	0.54–3.77	0.7	0.27–1.87	–	–	–	–	–	–	–	–	–	–
Whole milk with coffee and tea	0.85	0.64–1.12	1.18	0.89–1.57	–	–	–	–	–	–	–	–	–	–
Chicken	1 Time		2 Times		3 Times		4 Times		5 Times		6 Times		More than 6 Times	
Dark chicken meat without skin	1.25	0.81–1.94	0.77	0.47–1.25	0.95	0.36–2.52	–	–	–	–	–	–	–	–
Fried dark chicken meat with skin	0.51	0.26–1.01	2.02	0.97–4.19	1.6	0.23–11.11	–	–	–	–	–	–	–	–
Fried white chicken meat with skin	1.14	0.48–2.73	0.88	0.37–2.09	–	–	–	–	–	–	–	–	–	–
White chicken meat with skin	0.96	0.53–1.72	0.9	0.45–1.79	1.86	0.61–5.68	–	–	–	–	–	–	–	–
Fish	1 Time		2 Times		3 Times		4 Times		5 Times		6 Times		More than 6 Times	
Fat added fried fish	0.84	0.58–1.23	1.19	0.82–1.73	–	–	–	–	–	–	–	–	–	–
Oil packed canned tuna	0.62	0.35–1.08	2.07	1.12–3.81	–	–	–	–	–	–	–	–	–	–
Pork	1 Time		2 Times		3 Times		4 Times		5 Times		6 Times		More than 6 Times	
Pork	0.96	0.74–1.24	1.04	0.81–1.35	–	–	–	–	–	–	–	–	–	–
Bacon	0.94	0.80–1.11	0.96	0.78–1.20	–	–	1.22	0.92–1.62	1.19	0.66–2.15	–	–	1.11	0.58–2.12
Lean bacon	0.91	0.68–1.22	0.94	0.62–1.41	1.61	0.73–3.55	1.26	0.76–2.09	–	–	–	–	–	–
Regular bacon	0.98	0.81–1.19	0.9	0.69–1.17	0.9	0.34–2.37	1.28	0.90–1.83	1.29	0.62–2.69	–	–	1.08	0.45–2.59
No negative relation														
Food items servings per day	1 Time		2 Times		3 Times		4 Times		5 Times		6 Times		More than 6 Times	
Beef	RR	CI	RR	CI	RR	CI	RR	CI	RR	CI	RR	CI	RR	CI
Beef steak	1.4	0.67–2.92	0.72	0.34–1.50	–	–	–	–	–	–	–	–	–	–
Butter	1 Time		2 Times		3 Times		4 Times		5 Times		6 Times		More than 6 Times	
Reduced fat butter	1.39	0.73–2.67	0.72	0.37–1.37	–	–	–	–	–	–	–	–	–	–
Regular butter with pancakes/waffles	1.25	0.74–2.10	0.8	0.48–1.35	–	–	–	–	–	–	–	–	–	–
Regular butter with potatoes	1.14	0.99–1.31	0.82	0.67–0.99	1.03	0.72–1.46	0.93	0.71–1.22	1.01	0.66–1.54	–	–	–	–
Cheese	1 Time		2 Times		3 Times		4 Times		5 Times		6 Times		More than 6 Times	
Regular cheese	1.13	1.02–1.25	0.91	0.79–1.05	0.77	0.54–1.11	0.96	0.81–1.13	0.9	0.68–1.20	–	–	0.77	0.53–1.13
Milk	1 Time		2 Times		3 Times		4 Times		5 Times		6 Times		More than 6 Times	
Soy milk with coffee and tea	1.61	0.23–11.36	0.62	0.09–4.36	–	–	–	–	–	–	–	–	–	–
Chicken	1 Time		2 Times		3 Times		4 Times		5 Times		6 Times		More than 6 Times	
Dark chicken meat with skin	1.41	0.59–3.36	0.66	0.25–1.76	0.99	0.14–6.96	–	–	–	–	–	–	–	–
Dark fried chicken meat without skin	2.65	0.38–18.73	0.38	0.05–2.66	–	–	–	–	–	–	–	–	–	–
White fried chicken meat without skin	1.26	0.66–2.42	0.76	0.36–1.59	0.94	0.24–3.72	–	–	–	–	–	–	–	–
White chicken meat without skin	1.38	1.14–1.68	0.73	0.59–0.92	0.71	0.49–1.03	–	–	–	–	–	–	–	–
Moderate relation														
Food items servings per day	1 Time		2 Times		3 Times		4 Times		5 Times		6 Times		More than 6 Times	
Low fat ice cream and ice milk	1	0.83–1.20	1.09	0.86–1.37	0.47	0.21–1.05	1.13	0.79–1.62	0.8	0.38–1.66	0.92	0.42–2.04	–	–
Regular ice cream	0.89	0.79–1.01	1.07	0.91–1.25	0.92	0.61–1.39	1.35	1.10–1.66	0.94	0.67–1.33	–	–	–	–

‘–’, not applicable (no data/studies available to calculate RR, CI).

1 time—0 to 0.15 g, 2 times—0.16 to 0.3 g, 3 times—0.31 to 0.45 g, 4 times—0.46 to 0.6 g, 5 times—0.61 to 0.75 g, 6 times—0.76 to 0.9 g, > 6 times— ≥ 0.91 g.

### Positive relation

#### Alcohol

**Figure 2:**
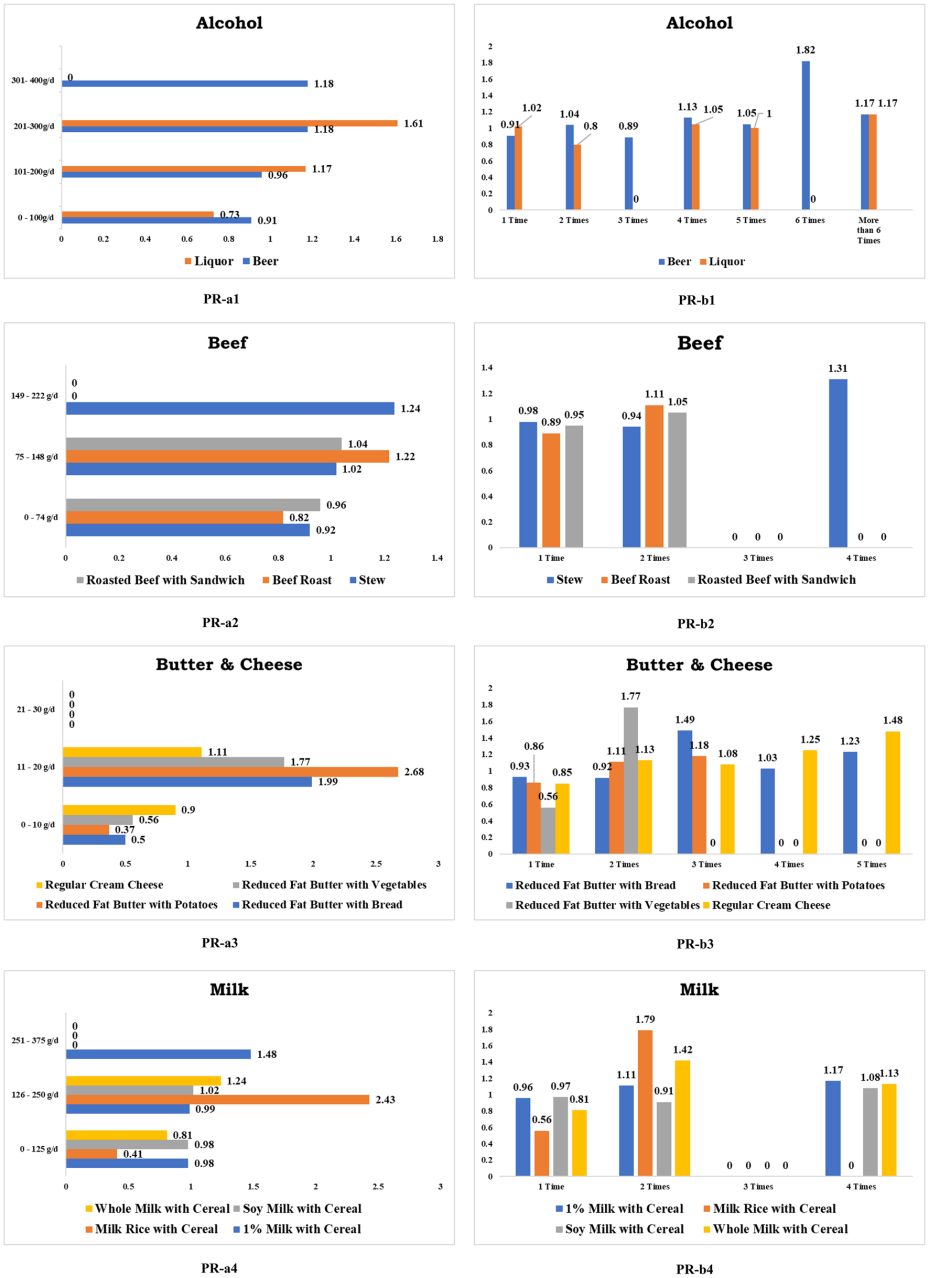
Foods positively related (PR) to colorectal cancer. *Note*: a1, a2, a3 & a4 denote daily consumption. b1, b2, b3 & b4 denote weekly consumption.

Any form of alcohol consumption is harmful to human health. Drinking more than 300 g of beer per day (RR 1.18, CI 0.90–1.56) and more than four times per week (RR 1.13, CI 0.87–1.47) were linked to an increased chance of getting CRC cancer. Likewise, having more than 200 g of liquor per day (RR 1.17, CI 0.77–1.78) and taking more than four times per week (RR 1.05, CI 0.82–1.34) were positively associated with an elevated CRC risk. Figure 2 shows some foods that promote CRC development.

#### Beef

Consuming more than 74 g of beef stew, beef pot pie, or beef mixtures daily (RR 1.02, CI 0.72–1.45) and having more than twice per week (RR 1.31, CI 0.71–2.43), remarkably enhances the CRC hazards. Overeating roasted beef over 74 g every day (RR 1.22, CI 0.68–2.19) and taking it more than once a week (RR 1.11, CI 0.95–1.30) substantially boosts CRC risk. Furthermore, eating sandwiches with roasted beef excess of 74 g per day boosts CRC danger (RR 1.04, CI 0.34–3.19) and having it more than once a week (RR 1.05, CI 0.77–1.43) considerably enhances CRC threat.

#### Dairy products

##### Butter and cheese

Consuming over than 10 g of reduced-fat butter on bread every day (RR 1.99, CI 0.91–4.39) and even more than two times a week (RR 1.49, CI 0.93–2.38) abnormally enhances CRC threats. Overconsumption of low-fat butter over 10 g with potatoes on a daily (RR 2.68, CI 0.69–10.42) and eating more than once per week (RR 1.11, CI 0.72–1.71) both raised the risk of CRC. Likewise, eating over than 10 g of low-fat butter with vegetables every day (RR 1.77, CI 0.26–12.27) and eating more than once a week (RR 1.17, CI 0.76–1.79) doubles the CRC danger. Additionally, an increased risk of CRC was confirmed for those who consumed more than 10 g of cream cheese made from milk and cream with other foods daily (RR 1.11, CI 0.68–1.81) and those who ate it more than once a week (RR 1.13, CI 0.82–1.55).

##### Milk

Consuming more than 250 g of low-fat milk mixed with cereal daily (RR 1.48, CI 0.56–3.91) and especially taking it more than once a week (RR 1.11, CI 0.83–1.48) increases the risk of CRC. Eating milk more than 125 g mixed with rice and cereal daily (RR 2.43, CI 0.36- 16.65) and taking it more than once a week (RR 1.79, CI 0.68–4.70) dramatically boosting CRC growth. Furthermore, ingesting more than 125 g of milk with soy cereal daily (RR 1.02, RR 0.33–3.14) and taking it more than twice a week (RR 1.08, CI 0.61–1.89) was adequate for accelerating CRC formation. Similarly, consuming more than 125 g of non-fortified milk with cereal daily (RR 1.24, RR 0.81–1.89) and dinning it more than twice a week (RR 1.42, CI 1.00–2.02) accelerates CRC risk.

#### Mixed foods

Some of the dataset’s food item values fall within the category of mixed foods, i.e., values that include values from more than one foodstuff. Various varieties of mixed food that can influence CRC incidence have been investigated. Generally, chili is known as beef chili because the chili food contains a significant amount of about 21% red meat, especially beef. Study findings confirmed that eating more than 74 g of beef chili per day (RR 1.39, CI 0.88–2.20), eating chili prepared only with beef (RR 1.64, CI 0.24–11.38), ingesting beef meatballs (RR 1.06, CI 0.67–1.68), and consuming beef stew mixtures (RR 2.37, CI 0.78–7.22) all facilitates the CRC growth. Furthermore, consuming dishes prepared from beef or pork liver more than once a week (RR 1.09, CI 0.41–2.89) significantly increases the CRC threat.

### Negative relation

#### Beef

**Figure 3:**
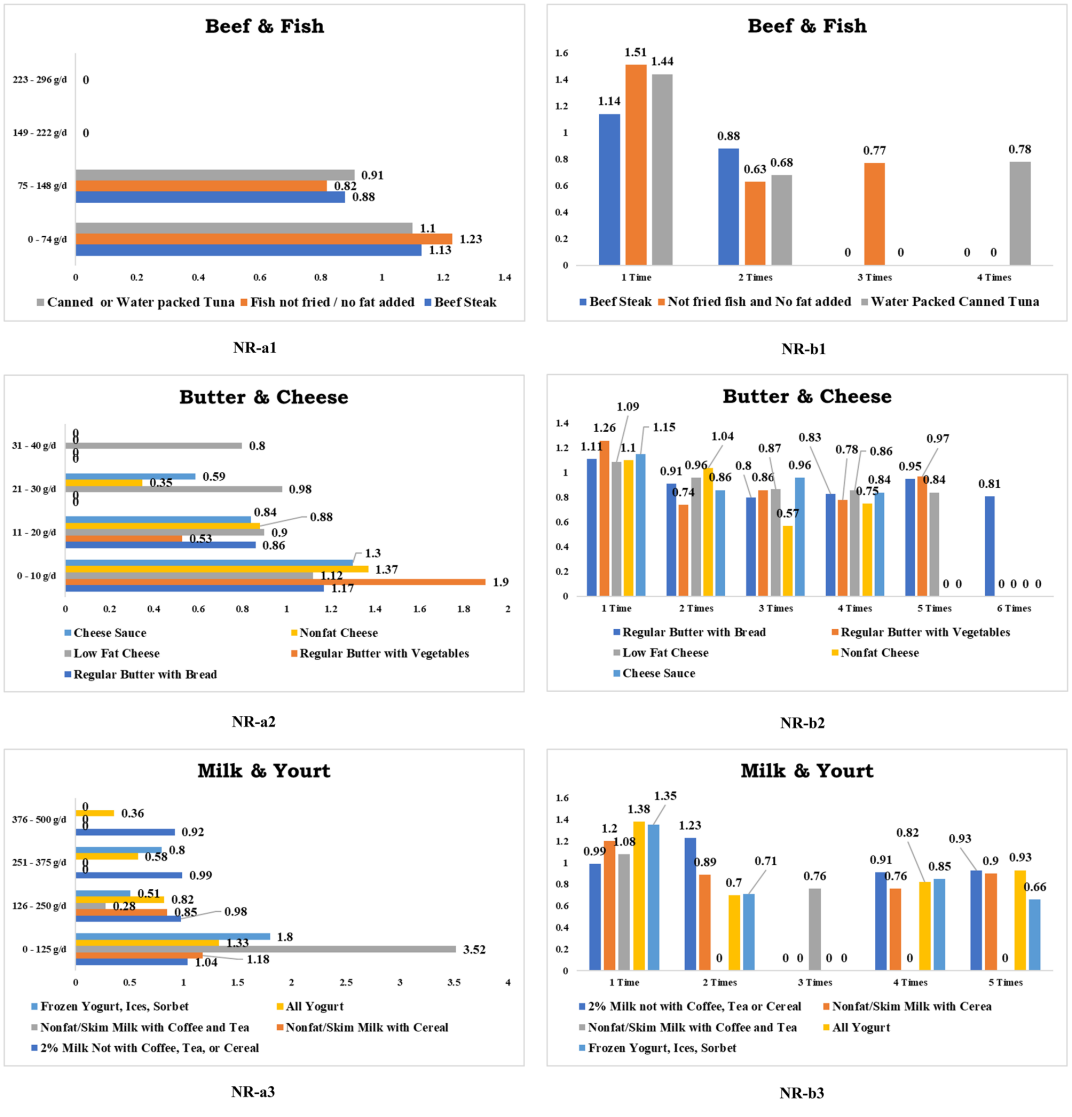
Foods negatively related (NR) to colorectal cancer. *Note*: a1, a2 & a3 denote daily consumption. b1, b2 & b3 denote weekly consumption.

All kinds of beef do not increase CRC risks. Conversely, consuming specific types of beef is linked to a decreased risk of CRC. Consumption of beef steaks in excess of 75 g daily (RR 0.88, CI 0.50–1.55) and having it more than once a week (RR 0.88, CI 0.62–1.23) significantly decreased the CRC hazards. Figure 3 depicts a few foods that reduce the CRC risk.

#### Dairy products

##### Butter and cheese

Regularly ingesting above 10 g of natural chemical-free butter on bread daily (RR 0.86, CI 0.58–1.26) and having it at least twice a week (RR 0.91, CI 0.76–1.08) safeguard us from CRC danger. Likewise, consuming more than 10 g of chemical-free butter with veggies each day (RR 0.53, CI 0.13–2.09) and dining it at least twice a week (RR 0.74, CI 0.57–0.96) decreases the formation of CRC and protects us. Daily consuming more than 10 g of low-fat cheese mixed with any foodstuffs (RR 0.9, CI 0.67–1.22) and specifically having it at least twice a week (RR 0.96, CI 0.79–1.18) significantly decreases CRC threat. Eating more than 10 g of non-fat cheese regularly in the daily diet (RR 0.88, CI 0.51–1.52) and eating it at least three times a week (RR 0.57, CI 0.26–1.28) decreasing the CRC risk. Furthermore, eating more than 10 g of cheese sauce daily (RR 0.84, CI 0.48–1.47) and having it at least twice a week (RR 0.86, CI 0.65–1.12) are linked to preventing CRC development.

##### Milk

In accordance with the study findings, consuming specific foodstuffs combined with value-added dairy products will protect from the threat of CRC. Daily consumption of more than 125 g of 2% fat milk with foods other than coffee, tea, or cereal (RR 0.98, CI 0.76–1.26) and eating soy milk above 125 g with foods other than coffee, tea, or cereal (RR 0.27, CI 0.04–1.91) both inhibits the development of CRC. Similarly, consuming more than 125 g of skimmed milk daily with cereal (RR 0.85, CI 0.66–1.09), drinking with coffee or tea (RR 0.28, RR 0.07–1.13), and eating mixing with other foods (RR 0.9, CI 0.71–1.14) all reduces the severity of CRC cancer. In terms of weekly intaking, consuming 2% fat milk with regular foods (excluding coffee, tea, or cereal) at least thrice a week (RR 0.91, CI 0.67–1.23) and eating soy milk with everyday foods (excluding coffee, tea, or cereals) more than twice in a week (RR 0.29, CI 0.07–1.15) was negatively correlated with CRC risk. Ingesting skimmed milk with grains or cereal more than once a week (RR 0.89, CI 0.71–1.10), drinking skimmed milk with coffee or tea more than twice a week (RR 0.76, CI 0.43–1.34), and eating skim milk with regular foods more than once a week (RR 0.83, CI 0.64–1.07) all decrease the likelihood of CRC formation. Surprisingly, eating over than 125 g of fresh yogurt (RR 0.82, CI 0.66–1.01) per day and having it at least twice a week (RR 0.7, CI 0.55–0.89) significantly decreases CRC risk. Similarly, consuming more than 125 g of frozen yogurt daily (RR 0.51, CI 0.24–1.07) and ingesting it twice a week (RR 0.71, CI 0.47–1.07) were both negatively associated with CRC formation.

#### Chicken

There are two varieties of chicken flesh: dark chicken meat and white chicken meat. Dark chicken flesh comes from chicken thighs and drumsticks (chicken legs), whereas white chicken meat comes from chicken breasts and wings. Daily consuming more than 74 g of dark chicken meat (RR 0.66, CI 0.09–4.66), white chicken meat (RR 0.24, CI 0.03–1.68), fried chicken mixtures (RR 0.86, CI 0.67–1.11), regular chicken (not in mixtures) (RR 0.2, CI 0.05–0.81), grounded chicken meat (RR 0.88, CI 0.12–6.14), and turkey meat (RR 0.43, CI 0.06–3.07) were all associated with minimization of the CRC hazards. Based on weekly consumption, consuming fried chicken mixtures more than twice (RR 0.93, CI 0.78–1.11), ingesting regular chicken meat twice (RR 0.91, CI 0.79–1.05), and eating turkey meat twice (RR 0.88, CI 0.59–1.32) connected with a lower CRC risk. In addition, consuming ground chicken more than three times (RR 0.56, CI 0.18–1.72) reduces the CRC danger.

#### Fish

Consuming more than 74 g of non-fat and non-fried fish daily (RR 0.82, CI 0.31–2.16) and having more than twice a week (RR 0.63, CI 0.46–0.86) were both linked with a lower risk of CRC. Similarly, dining daily more than 74 g of tuna (RR 0.73, CI 0.18–2.90), canned tuna (RR 0.91, CI 0.23–3.61), and having canned tuna twice a week (RR 0.68, CI 0.49–0.94) all associated with a lower risk of CRC.

#### Mixed foods

Hot dog is a non-vegetarian dish consumed with grilled or steamed meat inside two slices of bread. Eating more than 74 g of hot dogs per day (RR 0.68, CI 0.10–4.81) was connected to a lessen CRC danger. Lasagna, ravioli, and shell dishes are traditionally prepared with noodles, meats, cheese, and sauce mixes. Consuming more than 74 g of lasagna, ravioli, and shell foods per day (RR 0.68, CI 0.36–1.31) was associated with a lower risk of CRC.

### No positive relation

#### Beef

**Figure 4:**
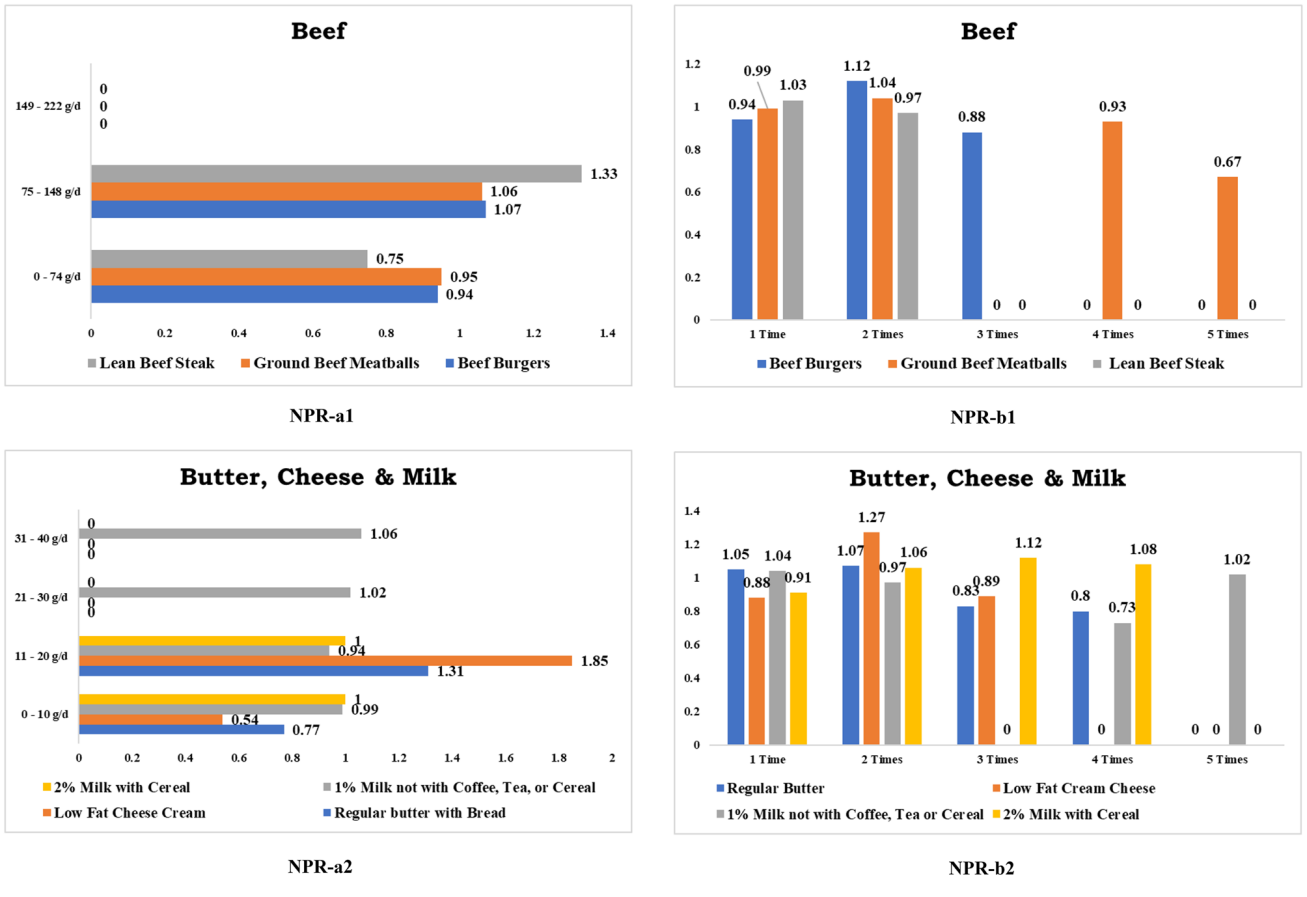
Foods not positively related (NPR) to colorectal cancer. *Note*: a1& a2 denote daily consumption. b1& b2 denote weekly consumption.

Consuming over than 74 g of roast beef with other food items, excluding sandwiches, reduces CRC risk (RR 0.99, CI 0.41–2.36), and eating at most once a week (RR 0.91, CI 0.62–1.34) considerably reduces CRC risk. Daily ingesting of fewer than 74 g of beef burgers (RR 0.94, CI 0.52–1.69), consuming burgers prepared with lean beef (RR 0.21, CI 0.05–0.78), taking burgers cooked with regular beef (RR 0.81, CI 0.26–2.49) and intake burgers almost once a week (RR 0.94, CI 0.80–1.11) all significantly decreasing CRC risk. Likewise, intaking daily lesser than 74 g of grounded beef or Meatloaf (RR 0.95, CI 0.60–1.50) and having it atleast thrice a week (RR 0.93, CI 0.60–1.44) reduces the CRC incidence. Similarly, eating fewer than 74 g of lean beef steak every day (RR 0.75, CI 0.36–1.56) and dining it twice a week (RR 0.97, CI 0.64–1.46) reduces CRC risks significantly. Figure 4 displays some foods that are not positively linked with CRC.

#### Dairy products

##### Butter and cheese

Every day ingestion of lesser than 10 g of regular butter with other foods, except bread (RR 0.77, CI 0.41–1.42), dining it atleast thrice a week (RR 0.83, CI 0.52–1.33), and eating reduced-fat butter with pancakes or waffles about once a week (RR 0.49, CI 0.21–1.17) significantly decreases CRC risk. Similarly, daily consumption of fewer than 10 g of cheesecake (RR 0.98, CI 0.80–1.20), intaking low-fat cream cheese (RR 0.54, CI 0.27–1.07), and eating low-fat cream cheese at least once a week (RR 0.88, CI 0.68–1.16) lowers the risk of CRC.

##### Milk

Consumption of less than 250 g of low-fat milk daily with coffee or tea (RR 0.98, CI 0.14–6.86) or with other foods (RR 0.94, CI 0.68–1.30) was related to a decreased risk of CRC. Intake of more than 125 g of cow’s milk with tea or coffee every day (RR 0.57, CI 0.14–2.26), drinking 2% fat milk with tea or coffee (RR 0.46, CI 0.15–1.43), and eating with other foods (RR 1, CI 0.80–1.26) significantly decreases the CRC hazards. Daily drinking less than 125 g of condensed milk with tea or coffee (RR 0.24, CI 0.04–1.63) reduces the risk of CRC. Consuming at least twice a week 1% low-fat milk with other foodstuffs excluding tea or coffee (RR 0.97, CI 0.68–1.39) and drinking condensed milk with coffee and tea (RR 0.7, CI 0.27–1.87), both linked with a lower risk of CRC. On the other hand, consuming 2% low-fat milk with tea or coffee once a week (RR 0.94, CI 0.78–1.14), drinking cow’s milk with coffee and tea (RR 0.85, CI 0.64–1.12), and ingestion of 2% fat milk with cereal (RR 0.91, CI 0.81–1.03) were all associated with a lower risk of CRC. Similarly, ingesting cow’s milk with food other than coffee and tea about once a week (RR 0.87, CI 0.70–1.08) is associated with a lower risk of CRC.

#### Chicken, fish and pork

Daily consumption of less than 74 g of non-fried skinless chicken dark meat (RR 0.4, CI 0.06–2.71), fried white meat chicken with skin (RR 0.44, CI 0.06–3.03), and eating non-fried skinless chicken dark meat more than twice a week (RR 0.77, CI 0.47–1.25), as well as having fried white meat chicken with the skin at least twice a week (RR 0.88, CI 0.37–2.09), all were reduces the chance of developing CRC. Intake of dark fried chicken meat with skin nearly once a week (RR 0.51, CI 0.26–1.01) and white chicken meat with skin at least twice a week (RR 0.9, CI 0.45–1.79) reduces CRC risks. Consuming more than 75 g of fat-added fried fish per day (RR 0.78, CI 0.32–1.86) and eating it once a week (RR 0.84, CI 0.58–1.23) reduces CRC risk significantly. Eating oil-packed canned tuna at most once a week (RR 0.62, CI 0.35–1.08) decreases CRC development. Daily eating more than 74 g of pork (RR 0.67, CI 0.17–2.65) and having it once a week (RR 0.96, CI 0.74–1.24) protects us from CRC attacks.

### No negative relation

#### Beef and poultry

**Figure 5:**
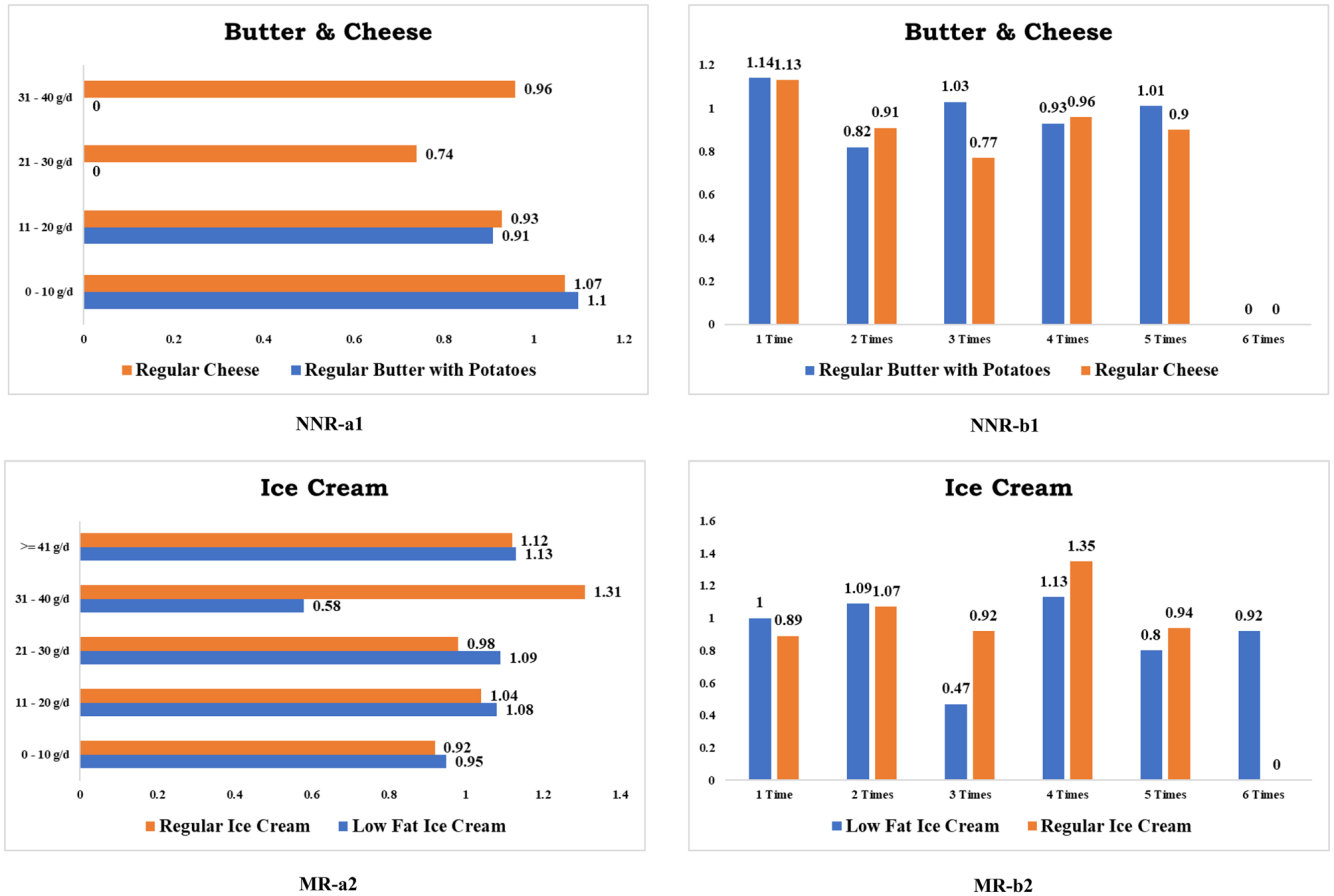
Foods no negatively related (NNR) & moderate related (MR) to colorectal cancer. *Note*: NNR and MR a1 denotes daily consumption. NNR and MR b1 denotes weekly consumption.

Dining regular beef steak at least twice a week (RR 0.72, CI 0.34–1.50) considerably minimizes the risk of CRC. Consuming dark meat chicken with skin more than once a week (RR 0.66, CI 0.25–1.76), eating dark meat fried chicken without skin (RR 0.38, CI 0.05–2.66), dining white meat fried chicken without skin (RR 0.76, CI 0.36–1.59), and taking white meat chicken without skin (RR 0.73, CI 0.59–0.92) all dramatically reduce CRC hazards. Figure 5 depicts some food items that are not negatively associated with CRC.

#### Dairy products

##### Butter, cheese and milk

Overconsumption of regular butter over 10 g with potatoes every day (RR 0.91, CI 0.48–1.75) and eating almost twice per week (RR 0.82, CI 0.67–0.99) both lower the risk of CRC. Reduced-fat butter eating with any dishes at least twice a week (RR 0.72, CI 0.37–1.37) and having regular butter with pancakes and/or waffles twice a week (RR 0.8, CI 0.48–1.35) significantly reduced CRC risk. Likewise, lessened CRC risk was confirmed for those who consumed more than 10 g of regular cheese every day (RR 0.93, CI 0.78–1.10) and those who ate it more than twice a week (RR 0.91, CI 0.79–1.05). Additionally, soy milk intake in coffee and tea almost twice a week (RR 0.62, CI 0.09–4.36) decreases CRC hazard.

### Moderate relation

#### Butter–ice cream and ice milk

In accordance with our results show that there was no definitive link between CRC cancer and the consumption of low-fat ice cream, regular ice cream, and ice milk. Figure 5 depicts some food items that are moderately related to CRC.

## Conclusions and future needs

The proposed research is based on food, aiming to find out what kind of food intake stimulates the cells that cause deadly diseases like CRC cancer. The study compares several machine learning models on a real-time dataset and identifies which algorithm performs best in accuracy and other performance metrics. Since the research used real-time information, the dataset had asymmetric input and output values. The SMOTE technology was used to handle these. The SMOTE model artificially augments the minority class data (input) to be equivalent to the majority class data (output). Then, all ML model accuracies were calculated before and after applying SMOTE to the dataset. Finally, after solving the imbalance problems using SMOTE, all ML models predicted the CRC patients more accurately. At the end of the study, the SMOTE-KNN classification algorithm performed well on a real-time dataset and identified CRC patients with high accuracy.

The proposed study examines the common foods individuals consume daily and various subtypes of those foods. Red meat intake, specifically beef stew, roasted beef, and sandwiches with roasted beef, revealed a positive connection with CRC risk, in line with previous epidemiological research^48–51^. Furthermore, our findings demonstrate that eating particular forms of beef, such as beef steaks, roast beef with sandwiches, beef burgers, ground beef or meatloaf, and lean beef steak in excess of 74 g daily and no more than once a week significantly lowers CRC risk. In accordance with these findings, it is clear that not all red meat consumption would increase the CRC hazards. Eating hot dogs, lasagna, ravioli, and shell foods has recently been widespread in many places. Our research also examines those foods under the mixed foods segment and finds that eating hot dogs, lasagna, ravioli, and shell foods dramatically reduces CRC. However, consuming beef meatballs and beef or pork liver elevated the CRC risk. Overall, our research findings reveal a complex link between red meat consumption and CRC risk, emphasizing the significance of including particular types and amounts of red meat in dietary recommendations.

The majority of the epidemiological research suggested that poultry intake mitigates CRC risk. However, in practical terms, most of us have the chance to taste multiple subtypes of chicken, like chicken wings, chicken legs, boneless chicken, chicken with skin, and skinless chicken etc. Therefore, in addition to focusing on normal chicken meat, it is essential to investigate different varieties to understand their impact on CRC risk better. Our study results indicate that daily consumption of over 74 g of dark chicken, white chicken, fried chicken, ground chicken, and turkey is negatively associated with CRC incidence. Eating in excess of 74 g daily of non-fat and non-fried fish, tuna, canned tuna, and pork was found to reduce the risk, which aligns with previous results^52,53^. Moreover, being careful about the amount while eating certain types of chicken can protect us from CRC. The findings reveal that consuming more than 74 g of non-fried skinless chicken dark meat and fried white meat chicken with skin on a daily basis promotes CRC risk. Rather than suggesting that all poultry ingestion enhances the CRC threat^54^, our finding clearly highlights which form of chicken varieties and to what limits may raise the risk.

Our findings reveal some crucial links between dairy products and CRC risk. Nowadays, we have seen lots of chemical-added products available in the market and ingestion by most individuals. To understand the complex relationship between dairy products and CRC risk, an investigation should focus on chemical-added products. The proposed research comprehensively examined dairy products and their different subtypes. Notably, our findings confirms that consuming natural chemical-free butter, low-fat and fat-free cheese, reduced-fat and skimmed milk, and fresh and frozen yoghurt reduces CRC, consistent with previous studies^55,56^ results. On the flip side, when examining the value-added byproducts, it exposes the shocking correlation that increased CRC while eating low-fat milk and non-fortified milk mixed with cereal, eating cream cheese, and ingesting reduced-fat butter with bread and vegetables. According to our findings, it is evident that natural dairy products like milk and yoghurt mitigate CRC risk, while caution should be posed when consuming certain value-added dairy products. We strongly believe that these findings will be helpful for individuals when choosing dairy products.

Our research has limitations as well as future demands. The study addressed some foodstuffs in the conclusion and discussion part under two headings: No Positive relation and No Negative relation. Researchers could not classify them as Positive or Negative associations due to the scarcity of values for such food items. As we all know, people cannot taste all kinds of foods in one day; in fact, it is impossible; thus, some food item values are not in the real-time dataset. Therefore, conducting an in-depth analysis of such foods in this cohort study was impossible. If adequate values of such food items are accessible in the future, researchers hope to extend the study results in-depth and more accurately.

## Data Availability

Data are available from the NCI upon reasonable request.
